# The Four Critical Priority Fungi According to the World Health Organization and the Hope for New Therapies: A Focus on Cell Wall Antifungal Targets

**DOI:** 10.3390/jof12030162

**Published:** 2026-02-25

**Authors:** Gabriel Davi Marena, Gabriela Corrêa Carvalho, Martha Helena Chaves Magalhães, Julia Marcondes Figueiredo, Danilo Henrique Ramos, Joshua D. Nosanchuk, Carlos Pelleschi Taborda

**Affiliations:** 1 Laboratory of Pathogenic Dimorphic Fungi, Department of Microbiology, Institute of Biomedical Sciences, University of São Paulo, São Paulo 05508-000, SP, Brazil; gabmardavi@gmail.com (G.D.M.); marthahcm@gmail.com (M.H.C.M.); julia.marcondes.figueiredo@alumni.usp.br (J.M.F.); danilo.cajuru94@gmail.com (D.H.R.); 2 Faculty of Pharmaceutical Sciences, Food and Nutrition (FACFAN), Federal University of Mato Grosso Do Sul (UFMS), Cidade Universitária, Campo Grande 79070-900, MS, Brazil; gabrielacarvalho57@yahoo.com; 3 Departments of Medicine (Division of Infectious Diseases) and Microbiology and Immunology, Albert Einstein College of Medicine, Bronx, NY 10461, USA; josh.nosanchuk@einsteinmed.edu; 4 Department of Dermatology, Institute of Tropical Medicine of São Paulo, Faculty of Medicine, University of São Paulo, São Paulo 05403-000, SP, Brazil

**Keywords:** critical fungi, World Health Organization, resistance, cell wall, new therapeutics

## Abstract

In 2022, the World Health Organization (WHO) released a list of four fungi identified as the most medically important global pathogens, resulting in *Cryptococcus neoformans*, *Candidozyma auris* (formerly *Candida auris*), *Aspergillus fumigatus* and *Candida albicans* being classified as the critical priority fungi. The purpose of this list is to encourage the prioritization of fungal research and public policies to strengthen its control and combat fungal diseases. Among the criteria used in the analysis by the WHO to determine these critical threat pathogens were numbers of deaths; annual incidence; current global distribution; trends in the last 10 years; hospitalization; complications and sequelae; preventability; access to diagnostic tests; evidence-based treatments; and antifungal resistance. Difficulties in treatment, including due to antifungal resistance, are a major factor in the morbidity and mortality of these fungi. The fungal cell wall plays a fundamental role in maintaining cellular architecture and contributing to fungal survival. Thus, new approaches targeting the cell wall have been and are being developed. This review article aims to bring together studies from the last ten years focusing on the development of new treatment alternatives targeting the cell walls of the four critical priority fungi and discussing their potential for combating these deadly fungi of worldwide clinical importance.

## 1. Introduction

In an era where there is much concern about infectious disease epidemics, remarkably limited attention has been given to research on fungal infections, particularly regarding the development of new therapeutic options, even though serious mycoses represent a crucial burden of disease globally today resulting in more than 1.5 million deaths every year [1,2]. A bibliographic survey conducted from 2000 to 2020 in SCOPUS^®^ associating the following keywords—“infectious diseases/virus/new formulations”, “infectious diseases/bacteria/new formulations”, “infectious diseases/parasites/new formulations” and “infectious diseases/fungi/new formulations”—highlights this neglect regarding the development of more antifungal formulation options, since 41% of the identified studies aimed to develop formulations for the treatment of viral infections, followed by 28% for bacterial diseases, 17% for parasite infections and 14% for mycoses [3].

Given the ongoing lack of attention to fungal diseases, the World Health Organization, in 2022, published a fungal priority pathogens list (WHO FPPL) with the aim of encouraging the prioritization of fungal research and public policies to strengthen their control and combat globally important mycoses. In this list, nineteen fungi were classified according to their priority (critical, high, and medium) based on ten criteria (numbers of deaths; annual incidence; current global distribution; trends in last 10 years; inpatient care; complications and sequelae; preventability; access to diagnostic tests; evidence-based treatments; antifungal resistance) which were weighted according to their importance. Notably, the antifungal resistance criterion was the most important one (38.5%), and this further reinforces the urgency in studying new treatment options, with special emphasis on the four fungi designated as critical priority: *Cryptococcus neoformans*, *Candidozyma auris* (previously classified as *Candida auris*), *Aspergillus fumigatus* and *Candida albicans* [4].

From this perspective, this review seeks to provide an overview of the main characteristics of these four fungi considered to be of critical priority by the WHO and to provide a detailed overview of noteworthy therapeutic targets found in their cell walls.

### 1.1. General Characteristics

*C. neoformans*, the number one species on the WHO FPPL, is one of the main pathogenic species of the genus *Cryptococcus*. Globally distributed, this fungus is found in abundance in soil contaminated by pigeon feces. Cryptococcal diseases are typically opportunistic infections in immunocompromised patients, particularly in the setting of AIDS patients, transplantation, and iatrogenic immunosuppression [5,6]. This pathogen initially infects the respiratory tract and can subsequently disseminate to pass through the blood–brain barrier and cause meningitis or meningoencephalitis, which is the most lethal manifestation of this fungus [7].

Despite the limited accurate global epidemiological data on *C. neoformans*, estimates of mortality include 28.4% in North America and Australia [5], 58.8% in Madagascar (Patients with HIV) [8] and 58.5% in Brazil [9]. The presence of several virulence factors plays a crucial role in this fungal pathogenesis [10,11]. Cell surface alterations that are important for central nervous system invasion include the presence of a capsule; d-mannitol production, preventing phagocytes from eliminating the infection; melanization, important for inhibiting phagocytosis in vivo; phospholipase production, important for intracellular replication; capsule enlargement and immunomodulation; and urease production [7]. 

The second WHO critical priority fungus is *C. auris*, which was first identified in 2009 and rapidly spread globally. A retrospective review of *Candida* spp. collections was conducted, and the oldest strain of *C. auris* dates back to 1996 in South Korea [12]. Based on its genome, *C. auris* has been classified into six different geographic clades. However, more clades are expected to emerge [13]. In 2024, 2 years after the publication of the WHO FPPL, genetic studies revealed significant differences between *C. auris* and other species of the genus *Candida* resulting its reclassification into the *Candidozyma* genus, which also now accommodates the formerly *Candida haemulonii* (CAH) clade members [14]. In addition to having strong skin tropism, *C. auris* easily spreads in healthcare settings, contaminating the environment, abiotic surfaces (including equipment) and other patients. Due to its ability to survive on diverse surfaces for long periods, it has a high outbreak potential and is increasingly identified as the causative agent of focal and systemic infections, especially in immunocompromised patients in hospital environments [15,16]. As it is a reportable condition in many countries, official epidemiological data can be observed in the literature, for example in 2023 the Los Angeles County Department of Public Health determined that the mortality rate within 30 days of this fungus for the period of January 2020 and March 2023 was 26% and 20% in patients in which the fungus was isolated from sterile or non-sterile sites, respectively. In these two groups, the attributable mortality was 17% and 1.9%, respectively [17]. The Centers for Disease Control and Prevention (CDC) observed a mortality rate for 30 days of 34% in a study carried out in 42 hospitals in the period 2017–2022 [18]. In Saudi Arabia the Ministry of Health hospitals did a survey involving 45 hospitals in the years 2020 to 2022 (3 years) and observed a higher mortality rate, 41.5% [19].

In addition to these high mortality rates, another extremely important factor that further aggravates *C. auris* disease is the fact that there is an extremely high rate of antifungal drug resistance, including to azoles, echinocandins, and polyenes, the main first-line antifungal agents [20]. In addition to its multidrug resistance, this fungus has other virulence factors such as cell adhesion to host cells, favoring biofilm formation; adaptation to environmental stresses, such as the ability to adapt to environments with high salt concentrations and the ability to grow at high temperatures (>40 °C); morphological plasticity that favors its rapid adaptation to environmental changes; production of enzymes capable of damaging host tissues, facilitating its dissemination and nutrients capture; production of hydrolytic enzymes capable of activating inflammatory mediators, and inactivating the action of antifungals and the immune response [20,21].

*A. fumigatus*, the third fungus listed as a critical priority, is a saprotrophic fungus that produces mycotoxins and the major human pathogen from the genus *Aspergillus*, which has about 378 reported species [22,23]. This fungus is widespread in outdoor environments such as soil, air (possibility of daily inhalation by humans), and decaying vegetation (recycling carbon and nitrogen). *A. fumigatus* can grow in extreme environments such as temperatures up to 70 °C, nutrient depletions and low levels of metal ions, and ultraviolet light [24,25]. The infections caused by this fungus range from allergy and colonization to acute life-threatening invasive infections. Invasive diseases occur mainly in patients with underlying risk factors, such as viral infections (such as COVID-19, which increases mortality to >50%), prolonged neutropenia, underlying hematologic malignancy, organ or stem cell transplantation, severe lung disease, liver cirrhosis, corticosteroid therapy, and being admitted to an intensive care unit [26].

Global epidemiological data on infections caused by *A. fumigatus* are scarce. In a worldwide systematic review of the literature from 2016 to 2021, it was observed that the 6-week mortality rates from invasive aspergillosis ranged from 31 to 36%. This value is higher for 12 weeks when caused by resistant strains (54.5% for voriconazole (VORI)-resistant strains vs. 30.7% for susceptible strains) [26]. In addition to the resistance to azoles, there are reports in the literature of *A. fumigatus* strains resistant to triazoles (pan-azoles resistance presumably due to the use of this class as a fungicide in agriculture) and amphotericin B (AmB) [24,27]. As it is a saprophytic fungus, several virulence factors are necessary to confer pathogenicity, such as the ability of conidia to adhere to human tissues; thermotolerance; stress response, such as growth capacity in hypoxic environments; strategies to acquire nutrients from the human host, like secretion of enzymes capable of degrading human tissues; ability to maintain the integrity of the cell wall in the face of dynamic changes (function and form) during adaptation to the human host; resistance, evasion, and debilitation of the human immune system [28].

The fourth WHO critical threat fungus is *C. albicans*. This fungus belongs to the *Candida* genus, which is composed of about 200 species [29]. *C. albicans* is a ubiquitous commensal fungus in humans that colonizes mucosal surfaces, such as the vagina, gastrointestinal tract and oropharyngeal cavity [30]. Although its usual relationship with healthy humans is as commensal, imbalances of a host (due to several factors such as changes in microbiota, immune system impairment and genetic or phenotypic variations in the strain) may lead to a range of diseases from localized superficial mucocutaneous to systemic invasive infections, which can be fatal [31,32]. As with the critical threat fungi mentioned above, *C. albicans* also does not have updated official epidemiological data. However, there are several epidemiological study initiatives in various parts of the world. For example, an observational cohort study carried out in Europe by the European Confederation of Medical Mycology (ECMM), determined a 7.7% mortality rate for candidemia caused by *C. albicans* [33]. In a study conducted in China (at a regional tertiary teaching hospital), the 30-day mortality rates for patients with *C. albicans* blood stream was 18.5% [34]. On the other hand, in a cohort study conducted in Brazil at a state hospital in Belo Horizonte from May to November 2020, a mortality rate of 76.3% was observed for patients co-infected with *C. albicans* and COVID-19 [35].

**Figure 1:**
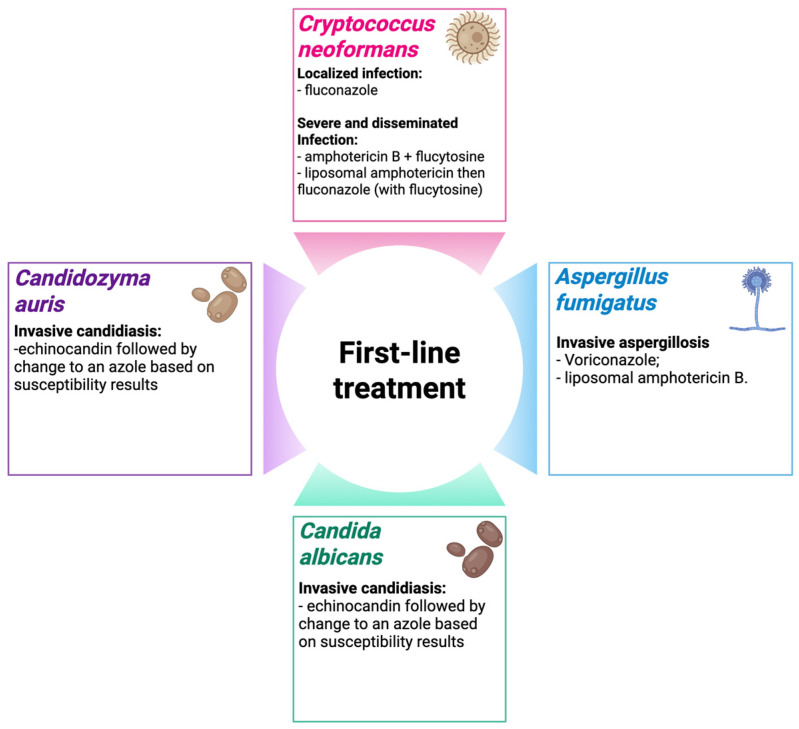
Comparative overview of first-line treatment for critical priority microorganisms according to the WHO [4]. Created in BioRender. Marena, G. (2026) https://BioRender.com/krr3d4x (accessed on 5 January 2026).

Azoles, especially fluconazole (FLU), have historically been the first-choice drug to combat this fungus; however, there is an increasing number of publications in the literature related to *C. albicans* strains resistant to FLU, miconazole and econazole [36]. In addition to the azole class, there are diverse reports about strains resistant to other drugs, such as the echinocandin class [37,38]. In addition to the development of resistance, *C. albicans* presents other virulence factors, such as the ability to change morphology, the pseudo-hyphal and hyphal forms are the invasive form of this fungus; secretion of proteins that gives it the ability to invade and adhere to the host and abiotic medical surfaces; biofilm formation; stress resistance, for example production of superoxide dismutases that convert O_2_ into hydrogen peroxide and molecular oxygen, freeing the fungus from oxidative death induced by the host’s immune system; and secretion of hydrolytic enzymes, which enable attachment to human tissue and lead to rupture of the host cell membrane [39]. Figure 1 summarizes the main treatment alternatives for fungal infections described in this work.

### 1.2. Fungal Cell Wall

The surface of a cell has two important roles: maintaining the integrity of the cell and interacting with the environment [40]. The cell wall of human pathogenic fungi represents approximately 40% of the total cellular volume and is of fundamental importance in maintaining cellular homeostasis. The critical nature of this is exemplified in yeast where 1/5 of the genome is devoted. The fungal surface is constantly exposed to changes such as temperature, osmotic pressure, pH, and other environmental and/or host factors. These changes cause stress to fungi resulting in rapid cellular modifications leading to alterations of their cell walls. Rebuilding, changing the architecture and composition, and repairing their cell walls is one of the adaptive methods practiced by fungi [41].

The cell wall is much more than just the outer layer of a fungus; it is a dynamic organelle capable of influencing fungal ecology as well as regulating it in response to stresses and environmental conditions. The dual interaction with the surrounding environment negatively or positively affects fungal survival, which is another characteristic of the cell wall. Furthermore, the cell wall plays a fundamental role in the pathogen–host interaction, since, for example, its composition contains the well-known pathogen-associated molecular patterns (PAMPs) recognized by pattern recognition receptors (PRRs) [42,43].

In addition to being eukaryotes, fungi have an ultrastructure similar to mammalian cells, which makes it difficult to develop antifungal options. However, some components present in the cell wall are not found in mammalian cells. In addition to providing mechanical resistance, the cell wall allows plasticity for growth and division, aiding in the formation of different types of cells throughout the life cycle. The main components of the cell wall are chitin, glucans and glycoproteins [44]. In most cases, the cell wall of fungi is made up of a layer composed of a more preserved skeleton (inner layer) and a layer with heterogeneous peculiarities and more specific to each species (outer layer) [42].

**Figure 2:**
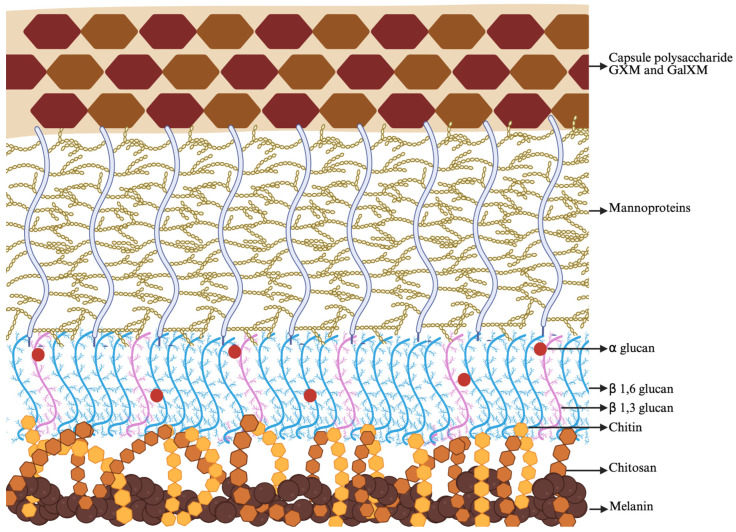
Schematic model of the cell wall of yeasts of the genus *Cryptococcus* sp. Created in BioRender. Marena, G. (2026) https://BioRender.com/jfrqmjh, (accessed on 5 January 2026).

The cell wall of *Cryptococcus* sp. is complex, composed mainly of glycoproteins, chitin, chitosan and glucans [45]. *Cryptococcus* sp. are yeasts that display on their complex cell wall a capsular structure composed of two polysaccharides, glucuronoxylomannogalactan (GalGXM ∼7 to 8% of the mass) and glucuronoxylomannan (GXM; ∼90% of the mass). There are also approximately 1% mannoproteins in the capsule. The polysaccharide GXM is a linear mannan linked to α-1,3 and GXMGal is an α-1,6-galactan [46]. Chitooligomers or chitin-derived oligomers, mannoprotein 84, 88, 98, 115, phospholipase B (Plb1) and mannoproteins (<1%) are among other components present in the cell wall of *Cryptococcus* sp. [47]. The capsule is a critical virulence factor for *Cryptococcus* sp. Importantly, the capsular polysaccharide is the target of current laboratory testing methods [48,49]. Chitosan also plays an important role in the virulence of *Cryptococcus* sp., and chitosan deficiency may cause alterations in the host response, inducing a protective immune response biased towards Th1 [50]. Another important component is melanin, synthesized from catecholamine precursors in exogenous tissue during infection and crucial to the virulence of the fungus, acting to increase the cells’ ability to resist elimination by the immune system [51]. Figure 2 shows carbohydrates, melanin, and mannoprotein that make up the cell wall and capsule of *C. neoformans*.

Chitin is a linear polymer of d-glucosamine linked to β-(1,4) and one of the main components present in cell walls of diverse living beings, especially fungal cells [52]. Significantly, chitin is absent from human cells, making it an important target for antimicrobial development. In *Cr. neoformans*, the chitin synthase/regulator system isoforms (Chs3 and Csr2) synthesize the majority of the chitin in the vegetative cell wall, which is subsequently converted into chitosan. Furthermore, this fungus has the capacity to express three chitin deacetylases, Cda1, Cda2, and Cda3, responsible for chitosan production. Strains lacking CHS3, CSR2, or all three chitin deacetylases exhibit altered chitosan production and, consequently, sensitivity to different cell wall inhibitors. Therefore, chitosan is essential for maintaining fungal integrity [51]. Another study also highlights the importance of chitin, especially chitosan, since chitosan-deficient strains were considered non-virulent and were rapidly eliminated from the lungs of mice infected with *C. neoformans* [53].

Secreted aspartic proteases, SAPs, are involved in the penetration of pseudohyphae into the host since they act in enzyme secretion capable of assisting in the adhesion, invasion, and destruction of factors of the host’s immune system, in addition to obtaining nutrients from the environment. Therefore, SAPs are related to fungal virulence [54,55]. May1 is one of the many proteases synthesized by *C. neoformans*, and its function is extremely important, acting mainly in tissue invasion, virulence, dissemination to the central nervous system, and nitrogen assimilation [56]. Mice immunized and producing antibodies against the SAP, Pep1p, showed greater survival, demonstrating that vaccination with Pep1p decreased the fungal load and stimulated the immune response. This highlights the importance of SAPs for the pathogenesis of *C. neoformans* [57].

*C. albicans* presents other virulence factors, such as the ability to change morphology, the pseudo-hyphal and hyphal forms. A difference between the morphologies is that the pseudohyphae and hyphal forms have significantly more chitin than yeasts. Furthermore, the structure of the mannans found in the cell wall differs between the morphotypes, with a significant decrease in mannan-oligosaccharides linked to β-1,2 phosphodiesterified acid when in the pseudohyphae form. However, the amount of side chains containing a stable β-1,2 bond to the acid does not change [58].

**Figure 3:**
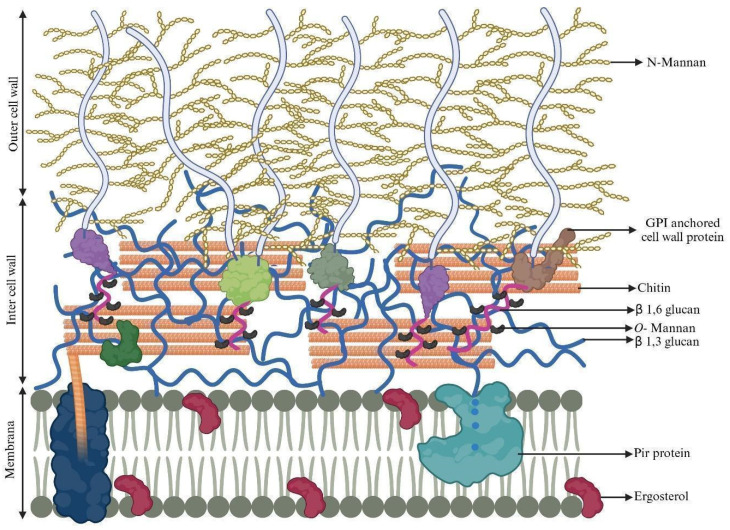
Schematic model of the cell wall of yeasts of the genus *Candida* spp. and *Candidozyma* sp. adapted from Lenardon et al. [59]. Created in BioRender. Marena, G. (2026) https://BioRender.com/8qee82n, (accessed on 5 January 2026).

Lenardon et al. [59] presented a refined structural model of the *C. albicans* cell wall. The inner layer of the wall is composed of chitin microfibrils interspersed with single and triple helices of (1,3)-glucan. Monosylated proteins are distributed throughout the inner wall (in greater proportion) and in the cell membrane (in lesser proportion). The glycosylphosphotidylinositol (GPI)-anchored proteins in the cell wall have a stalk region to which short O-mannans are attached. These GPIs include the agglutinin-like sequence (Als3, Als9, for example) secreted aspartyl proteinases (Saps), superoxide dismutase (Sod), endo-glucanase (beige), exo-glucanase, and chitinase. These proteins are linked to β(1,3)-glucan and to chitin via β(1,6)-glucan linkages (inner membrane region). Other proteins such as Pga, Eap1, Cht2, Pga7, Pga13, Pga26, Ecm33, Hwp1, Hwp2, Phr1, Plb5, Rbt1, Rhd3/Pga29 and Utr2 are also covalently linked to β(1,6)-glucan via GPI and are, as with the previous ones, called GPI-CWPs. Other non-GPI proteins (Int1, Bmh1, Sun41, and Mp65) and moonlight proteins (Eno1, Cdc19, Adh1, Hsp21, Hsp70, and Hsp90) are also present. The less abundant proteins, Pir (turquoise) are covalently linked to β(1,3)-glucan. Ergosterol is present in the cell membrane, one of the main components, and the main therapeutic target of polyenes (Figure 3) [59,60].

In *C. albicans*, chitin is synthesized by several distinct enzymes, Chs1 (class II), Chs3 (class IV), Chs2, and Chs8 (class I). In combination, these enzymes produce chitin in growth regions such as polarized budding tips, septations, and pseudohyphae [61]. The presence of chitin in the cell wall helps maintain the architecture of the cellular structure facilitating survival. In response to antifungal targeting of glucans, fungi increase the production of chitin to compensate for alterations in these complex carbohydrates, which is called the “cell wall rescue response” [62].

Agglutinin-like Sequence, ALS, genes are part of the adhesin family and are of particular interest as targets for new therapeutic alternatives since they are associated with adhesion, filamentation, biofilm formation and virulence of *Candida* and *Candidozyma* species. Als proteins consist of a highly complex N-terminal domain responsible for mediating protein-ligand interactions with host cells, a threonine domain, a central domain of less complexity with high variation in length and a C-terminal domain that anchors the adhesin to the fungal cell wall through a glycosylphosphatidylinositol anchor [63,64,65]. Als have eight distinct loci (Als1 to Als7 and Als9) and, among the loci, the main target, Als3, stands out, which when deleted most dramatically reduced the adhesion capacity of *Candida* [63,66]. Singh et al. [67] identified three proteins in *C. auris* isolates that presented sequence and structural homology to the Als3 protein of *C. albicans*. Another study detected a new specific adhesin in *C. auris* called Surface Colonization Factor (SCF1), which is essential for adhesion to biotic and abiotic surfaces. SCF1 aids in the adhesion of *C. auris* by promoting the interaction of cationic proteins with hydrated surfaces. Rich in cationic and aromatic residues, SCF1 can adhere to phosphatidylcholine, an electronegative component found in the cell membrane of mammals [68]. *C. auris* not only colonizes the skin surface, but also the dermis and persists for an indefinite period. Even with preventive measures, such as chlorhexidine gluconate, a study reports the concern that the use of this drug may deplete the microbiota and cause resistance among colonized pathogens [69].

The SAPs are encoded by a family of approximately 10 genes in *C. albicans* (SAP1-10), with Sap1-6 being studied most intensively [70,71]. A study points to evidence of the importance of SAP with pathogenicity of *C. auris*, although there is a divergence between the SAP of *C. auris* and the SAP of *C. albicans*, which can be justified by the variation between species [72].

Superoxide dismutases, SODs, are present in several pathogenic fungi, including *C. albicans*, acting against the oxidative burst of the host immune response. Much of what is known about SODs is associated with studies of SOD5 of *C. albicans*. *C. albicans* SOD5 is especially induced in pseudohyphae, a fundamental structure for tissue invasion, which contributes to the virulence of the pathogen [73]. SOD is an antioxidant enzyme involved in the synthesis of superoxide anions. Furthermore, SOD can be classified as copper and zinc (CuZnSOD), iron (FeSOD), nickel (NiSOD) or manganese (MnSOD) [74].

The *A. fumigatus* cell wall is a complex and dynamic structure composed mainly of carbohydrates, with varied composition according to the morphotype and to the environment pressure. *A. fumigatus* conidia are considered inert and have a double-layered cell wall. Internally, these conidia are composed of β-1,3-glucan, α-1,3-glucan and chitin, while the outer layer is composed of melanin and a rodlet layer. The two outer layer compounds are related to hydrophobicity, contributing to conidia air dispersion [43,75]. β-1,3-glucan is cross-linked to galactomannan, α-1,3-glucan and galactosaminogalactan and a single mixed molecule of β-1,3-1,4-glucans covalently [58].

Conidia germination involves losing the rodlet layer, reducing cell-wall thickness and exposing alpha-1,3-glucans to the outer environment. This increases the fungus’ ability to adhere and invade tissues. In its hyphal form, *A. fumigatus* cell wall is monolayered, with a central core composed of branched β-1,3-1,6-glucan attached to chitin and an amorphous structure of alpha-1,3-glucan and galactomannan [43]. Rodlets are located on the surface of conidia (composed of hydrophobic RodA proteins). This composition prevents conidia from being recognized by the host immune system. However, after conidia germination, the rodlet layer and melanin (another surface component) disappear, exposing α-(1,3)-glucans on the surface of swollen conidia [75].

This fungus does not have proteins covalently attached to the cell wall carbohydrates. The GPI-anchored proteins found in *A. fumigatus* do not have a structural role and, therefore, no covalent bonds are found, in contrast to *Candida* species [76]. Considering the cell wall composition, the main targets for *A. fumigatus* are related to polysaccharides.

**Figure 4:**
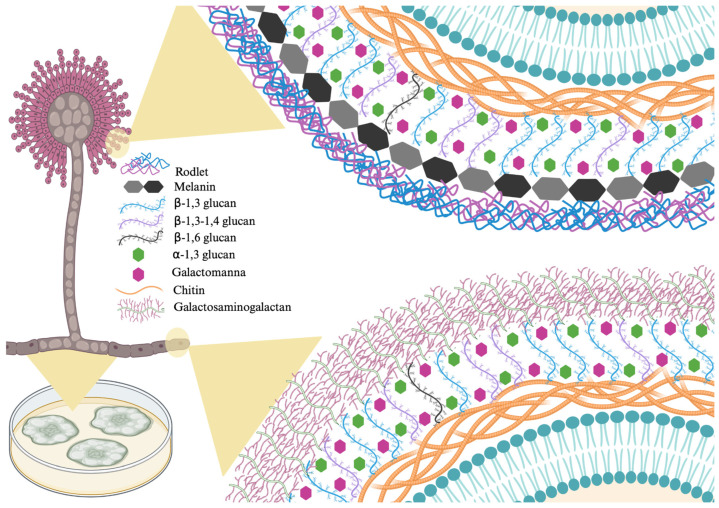
Schematic model of the cell wall of yeast forms of the genus *Aspergillus* sp. Created in BioRender. Marena, G. (2026) https://BioRender.com/qchthoe, (accessed on 5 January 2026).

On the other hand, the cell wall plasticity confers adaptive responses in this filamentous fungus. The use of echinocandins in aspergillosis treatment, targeting beta-1,3-glucan synthesis, is efficient in reducing this polysaccharide. In contrast, there is an increased amount of chitin and α-1,3-glucan, indicating a compensatory mechanism in the cell wall structure. This phenomenon is related to integrity maintenance and, therefore, treatment efficacy impairment, reinforcing the need of new antifungal drugs and constant attention to resistance [77]. Figure 4 shows the main proteins that make up the cell wall of *A. fumigatus*.

A group of researchers studied the influence of fungal allergens, Asp f 5 (matrix metalloprotease) and Asp f 13 (serine protease), on the inflammatory process, remodeling, and hyperreactivity of the airways in a mouse model. According to the results, proteolytic allergens, especially Asp f 13, contributed significantly to the airway and systemic inflammatory process in mice. Therefore, the important role of the serine protease Asp f 13 in asthma pathology, as well as that of the matrix metalloprotease allergen Asp f 5, is concluded [78].

## 2. Cryptococcus Neoformans

### 2.1. New Treatment Options: Nanomedicine and Drug Combinations

Silver nanoparticles (SNPs) obtained from the fungus *Fusarium oxysporum*, which can reduce aqueous silver ions extracellularly generating the SNPs, were tested by Ishida and collaborators [79], as a potential antifungal agent against *C. neoformans*. Firstly, the SNPs were produced and characterized, and then the fungi were exposed to the solution and MIC value of the nanoparticle was obtained according to the CLSI protocols, resulting in values ≤ 1.68 µg/mL, which was considered a susceptible concentration against *C. neoformans*, proving its potential antifungal effects. When the morphological features of the yeast cells treated with the SNPs’s sub-inhibitory concentration were microscopically evaluated, there were clear alterations of cell wall and capsule, that, according to previous studies, are the principal target of SNPs activity as an antimicrobial. The SNPs caused *C. neoformans* cell wall disruptions, serial invaginations, and increased thickness, highlighting the harm and alterations of the fungal cell surface generated by these molecules. Also, SNPs were retained on the capsule, highlighting the direct action of the particles on the cell wall and pointing to possible mechanisms of action of the SNPs.

Peng et al. [80] explored whether verapamil (VER), a calcium channel inhibitor drug widely used in hypertension, could be repurposed for use against *C. neoformans* alone and in combination with caspofungin (CAS). Although VER proved to be fungistatic, it was synergistic with CAS. Notably, VER reduced capsule size, and this was more pronounced in combination therapy, and the drug also caused aberrant cell appearances and non-detachment of buds. In vivo assays in *G. mellonella*, it was demonstrated that combination VER and CAS therapy resulted in a significant increase in survival and reduction in fungal burdens. VER treatment also downregulated chitin and glucan synthesis. The effects were attributed to VER effects on cellular calcium regulation that led to increased CAS susceptibility. Therefore, this combined therapy could be promising in cryptococcosis treatment. It is surprising that an antihypertensive drug possesses antifungal activity and, according to the authors, VER may affect the chitosan content present in the fungal cell wall, which justifies its antimicrobial activity. Furthermore, the antihypertensive drug acted by blocking calcium channels, reducing Ca^2+^ by inhibiting calcineurin, weakening the fungal cell wall and membrane.

### 2.2. Fosmanogepix and Cryptococcus neoformans

Shaw et al. [81] investigated a novel antifungal in clinical development, APX001A and its prodrug APX001, against *C. neoformans*. In addition, three closely analogous molecules, APX2020, APX2039, and APX2041, were tested in synergy with FLU. Fosmanogepix’s mechanism of action is the inhibition of the initial step of the GPI-anchored biosynthesis pathway by targeting glycosylphosphatidylinositol-anchored wall transfer protein 1 (Gwt1) [82]. Shaw et al. [81] found that APX001A resulting in an MIC of 0.25 μg/mL for *C. neoformaans*; APX2020 and APX2041: 0.031 μg/mL; and APX2039: 0.008 μg/mL. When tested against FLU-resistant strains (MIC ≥ 16 μg/mL), all APX molecules produced MICs in the susceptible range and APX2039 also exhibited the lowest MIC, ranging from 0.004 to 0.031 μg/mL in all strains evaluated. In a murine model, a significant decrease in CFU was reported in lung tissue in all groups. In the brains of *C. neoformans* infected mice, a statistically significant decrease in CFU was reported only in the combination-treated group and in the FLU group.

### 2.3. Natural Products

Kopecka et al. [83] studied how and why the latrunculin A (LA), a marine sponge toxin and inhibitor of microtubules and actin, affects the *C. neoformans* yeast cells. Cidality was detected within 20 h of LA treatment. Treatment led to aberrant cell walls formations with unusual thickness that were penetrating into cytoplasm, leading to a loss of viability, which highlights that the F-actin cytoskeleton essential for the *C. neoformans* cell wall function and formation.

The properties of bioactive molecules obtained from plants as antimicrobial agents are widely valued [84]. In this context, Cardoso et al. [85], explored the anti-*C. neoformans* activity of the essential oil of *Ocimum basilicum* var. Maria Bonita (EO); of its major compounds, linalool and geraniol; and of its synergism effect with fluconazole. Both MIC value assay and toxicity to mammalian cells assay were performed on the compounds. The MIC values for the EO, geraniol, linalool and fluconazole were, respectively, 1250, 76, 790 and 31.25 μg/mL against the strain tested. The CC_50_ toxicity for geraniol, EO and linalool were, respectively, 380, 310 and 197 μg/mL. When the fractional inhibitory concentration index (FIC) was calculated to evaluate if there was synergism between the compounds, synergy was demonstrated for the combinations between linalool plus geraniol (MIC reduced to 111 and 19 μg/mL, respectively) and geraniol plus fluconazole (MIC reduced to 19 and 4.14 μg/mL, respectively), which are concentrations compatible with safety based on the cytotoxicity assays. When the mechanism of action was evaluated through multiple tests, they found ergosterol synthesis inhibition rates in all strains treated with the compounds, ranging from 25 to 79%, in comparison with the control, indicating a possible pathway targeted by the compounds. The capsule was also affected by all the substances, presenting disruptions and considerable reduction in its thickness.

Horn et al. [86] investigated the principal component of *Cyperus rotundus*’s essential oil, α-Cyperone, as a potential antifungal agent against *C. neoformans*. The MIC value of this substance was high, 500 μg/mL, against the fungus. However, α-Cyperone and FLU was synergistic against *Pichia kudriavzevii,* resulting in 16-fold and 8-fold reductions in the MIC value of both FLU and α-Cyperone, respectively. The combination was not tested against *C. neoformans*. The effect of α-Cyperone on *C. neoformans* was evaluated and 16 µg/mL of α-Cyperone, resulting in a significant reduction in cell size. A cell wall disruption experiment revealed that 80% of *C. neoformans* of the treated group were destroyed following vortexing with beads, while only 50% of the control group were broken, indicating a possible weakening of the cell wall of the treated cells.

### 2.4. Other Molecules and Vaccines

Ferreira and collaborators [87] studied an antimicrobial peptide (AMP), the cationic peptide Ubiquicidin (UBI), which is found associated with the mammalian immune systems and well known for its antimicrobial properties. The UBI skeleton can be structurally modified, resulting in various derivatives with potent antifungal properties. Ferreira et al. produced and characterized different UBI derivatives compounds for testing against *C. neoformans* strains demonstrating MIC values ranging from 0.99 to 0.09 μmol/mL. The hypothesis of the peptides mechanism of antifungal action proposed in this study is based on the fact that AMPs are positively charged and can interact and bind to the fungus cell wall and capsule, since many molecules which constitute these regions, such as mannoproteins, glucuronoxylomannan and galactoxylomannan, are negatively charged, and these interactions can disrupt the stability and function of the region.

Hartland et al. [88] analyzed a library from the Broad Institute, composed of 361,675 molecules, to identify compounds active against *C. neoformans*. They screened for molecules that led to the release of adenylate kinase as a marker for cell lysis. Molecules that demonstrated mammalian cytotoxicity were eliminated. Next, they identified compounds that targeted the cell wall and performed MIC testing on the resulting molecules. This process identified benzothioureas molecules with MIC values ranging from 4 to 32 μg/mL. Mechanism of action studies indicated that inhibition of the signaling factors of the cell wall integrity pathway (CWIP), such as MAP (Mitogen-Activated Protein) kinase cascade, were responsible for the molecule’s effects against *C. neoformans*.

In the scenario of searching for new potential antifungal drugs against *C. neoformans* in molecules libraries, in 2017, Mayer et al. [89] screened the Pathogen Box, a library from the project Medicines for Malaria Venture (MMV, Switzerland) that contained 400 synthetic molecules with low mammalian cytotoxicity. *C. neoformans* growth was evaluated against all the compounds at low concentrations of 1 μL/mL. This identified MMV688271, a novel compound that completely inhibited fungal growth, even at 37 °C, with an MIC of 0.12 μg/mL. Microscopy studies revealed that cells exposed to the compound presented an aberrant appearance, suggesting that the mechanism of action may be associated with oxidative stress and cell surface integrity, and a sorbitol assay supported this hypothesis. Notably, a genetic assay revealed that mutants with a defect in transcription factor Nrg1, that encodes cell wall functions such as chitin synthases, had reduced susceptibility against MMV688271, further supporting a stress response mechanism.

In 2020, Ma et al. [90] investigated a cationic antimicrobial peptide (AMP), MSI-1, with stability against different stresses and low toxicity associated with mammalian cells. The MIC of the peptide against *C. neoformans*, including FLU-resistant strains ranged from 8 to 16 μg/mL. A murine infection model revealed that treatment with MSI-1 led to higher survival rates and significantly lower CFU of the brain and lung tissues. Also, there was a decrease in the production of inflammatory molecules in the treated group, showing an effective relief of fungal burden. Fluorescence microscopy analysis revealed a colocalization and association between FITC-MSI-1 and GXM of the capsule, consistent with the charged MSI-1 binding with the negatively charged GXM. Measurement of *C. neoformans* membrane integrity revealed that the treatment led to disruption of membrane integrity. Therefore, it was concluded that interaction of MSI-1 with GXM caused an imbalance that allows the insertion of the peptide into the lipid bilayer membrane of the fungus, increasing its permeability and fluidity and resulting in disruption.

De Oliveira et al. [91] also studied the Pandemic Response Box and found that MMV1593537 had an MIC range of 0.635 and 5 μg/mL against different *C. neoformans* strains, and fungicidal concentration activity of 10 μg/mL. The effect of the molecule was evaluated by electronic microscopy, revealing a significant reduction in the capsule and inhibition of budding as buds remained attached to their progenitor cells. Therefore, the researchers decided to investigate the effects of MMV1593537 on chitin and found increased chitinase peaks. Fluorescence microscopic assays demonstrated an increase in cell wall chitooligomers in cells exposed to MMV1593537, supporting that the molecule targets processes associated with chitin.

In 2022, a novel indolyenepheneethylene compound, EIPE-1, was synthesized by Adhikari et al. [92], and found to be effective against many Gram-positive bacteria, including methicillin-resistant *Staphylococci* strains. Its chemical structure was projected to act by disrupting cell-wall compounds. Conn et al. [93] subsequently investigated EIPE-1’s activity against *C. neoformans* and found an MIC range of 1.56 to 3.125 μg/mL against laboratory and clinical strains. Assays to evaluate the effect of the compound on the fungal cells were performed, revealing clear structurally aberrant cell walls and membranes in the treated group. RNA sequencing revealed that EIPE-1 affected many processes in *C. neoformans*, especially with capsule and ergosterol biosynthesis and cell-wall attachment and remodeling. However, in vivo assays with *G. mellonella* did not reveal differences in survival rates between EIPE-1 and untreated larvae.

Li et al. [94] explored the capsule as an adjuvant for a cryptococcosis vaccine which was based on mRNAs that encoded the antigen CDA1 (chitin deacetylase 1) and were packed inside LNPs. For this study, mRNAs encoding the CDA1 gene were synthesized and LNPs produced and characterized. To evaluate their efficacy, in vivo experiments with murine models were performed and mice were immunized with the CDA1-LNPs. However, no mice survived past 26 days post-infection. Next, cryptococcal capsules were obtained, purified, and added as an adjuvant with the CDA1-LNPs, resulting in a considerably stronger effect with 80% of the immunized mice surviving until the end of the experiment, 40 days post infection. Fungal burdens of surviving mice were also significantly lower in the CDA1-LPN group. Sera from the immunized mice that survived also revealed the generation of antibodies specific to CDA1. Table 1 summarizes all studies evaluated in this work against *C. neoformans*.

## 3. *Candidozyma auris*

### 3.1. New Treatment Options: Nanomedicine and Drug Combinations

Marena et al. [95] developed a coencapsulated nanoemulsion with amphotericin B (AmB) and micafungin (MICA). The results showed that the coencapsulated nanoemulsion significantly reduced the fungal load in the lungs, thymus, kidneys, spleen, and liver in a shorter treatment time when compared to free or separately nanoencapsulated drugs.

In another study, Marena et al. [96] explored the benefits of nanoemulsions of cholesterol and sunflower oil as a carrier for MICA (NEM) and evaluated its activity in in vivo and in vitro models using free MICA as a control. They demonstrated that NEM was more efficient in mature biofilms than the free drug. Testing in *Galleria mellonella* showed that the nanoemulsion potentiated the activity of MICA since NEM achieved a greater reduction in the fungal load compared to MICA.

### 3.2. Natural Products

Liu et al. [97] explored the in vitro efficacy of traditional herbal monomers (THMs) in relation to their antifungal activity. The THMs (*sodium houttuyfonate* (SH), *berberine* (BER), *palmatine* (PAL), *jatrorrhizine* (JAT), and *cinnamaldehyde* (CIN) act on the fungal cell wall on β-glucan and chitin interfering with their synthesis and transport, causing a compromised cell wall. The MIC results of the THMs separately ranged from 50 to 64 mg/mL, which is relatively high. However, a combination of compounds improved the MICs with the CIN/PAL resulting in an MIC of 12.5 mg/mL. The combinations induced damage to the fungal cell wall as demonstrated by significant alterations to the cell structure.

Tran et al. [98] studied the effects of the essential oils of cinnamon leaf and bark (CEOs against *C. auris* using direct and vapor diffusion. *Cinnamomum zeylanicum Blume* bark essential oil (CBEO) and *Cinnamomum zeylanicum Blume* leaf essential oil (CLEO) were tested. The MIC and CMFs of CBEO were lower than 0.03% (*v*/*v*), while CLEO showed 0.06% (*v*/*v*) and 0.25% (*v*/*v*) for MIC and CMFs, respectively. A recent study suggested that the active compound in CBEOs (*cinnamaldehyde*) compromises the fungal cell membrane and integrity of the cell wall [99].

Honorato et al. [100] demonstrated that the alkaloids solenopsins from fire ants were potent against *C. auris.* The solenopsins disrupted the membrane integrity of *C. auris* and the compound was effective against both the planktonic form and biofilm forms of the fungus. Furthermore, a synergistic effect between AmB and the alkaloid was demonstrated, even for AmB-resistant *C. auris* strains. Potent protective effects were also observed in a *G. mellonella* infection model.

Chauhan et al. [101], identified the enzyme *GCN5 lysine acetyltransferase*, which is essential for the remodeling and maintenance of fungal cell wall architecture, drug resistance and virulence, as a potential antifungal target. A *C. auris gcn5Δ* mutant was found to have a significant increase in exposed cell surface β-glucan and chitin content was increased. Given this characteristic, the authors then used the *lysine acetyltransferase* inhibitor *cyclopentanone, 2-[4-(4-chlorophenyl)-2-thiazolyl] hydrazone* (CPTH2) to evaluate whether disruption of this pathway affected fungal growth. Although no significant growth inhibition was detected with CPTH2 alone, its combination with CAS resulted in a marked reduction in growth.

### 3.3. New Antifungal Drugs

Hager and Larkin et al. [102] conducted in vitro and in vivo studies to evaluate the antifungal activity of fosmanogepix against *C. auris*. The in vitro results showed potent activity against all clinical isolates, with MIC_50_ values of 0.004 μg/mL and MIC_90_ of 0.031 μg/mL, lower than those observed for other antifungals, including anidulafungin, flucytosine, CAS, MICA, itraconazole (ITRA), posaconazole, VORI and AmB. In vivo, fosmanogepix significantly reduced the fungal load in kidney, lung and brain tissues. Berkow and Lockhart [103] also evaluated the efficacy of fosmanogepix against *C. auris* in an in vitro study with 100 clinical isolates from different countries. The MIC ranged from <0.005 to 0.015 mg/L, with no significant difference between clades and resistant isolates. Vázquez et al. [104] conducted a phase 2 clinical study with hospitalized patients in South Africa with *C. auris* candidemia, and treatment with fosmanogepix led to negative blood cultures in all patients who completed the study, demonstrating its safety and efficacy.

The novel compound turbinmycin was recently characterized from *Micromonospora* bacteria isolated from a sea squirt’s microbiome. Yeast phosphatidylinositol transfer protein (Sec14p) has been identified as the primary target of turbinmycin. Chemogenetic data at each concentration revealed DAmP (Down-of-Abundance Perturbation mRNA) mutants were highly sensitive to turbinmycin, with Sec14p being the most notable. Sec14p regulates the secretory function of the Golgi apparatus. Mice infected with *C. auris* and treated with turbinmycin showed dose-dependent antifungal efficacy with a logarithmic reduction of 3.610 (compared to control mice) at the highest dose [105].

Ibrexafungerp, a first-in-class triterpene antifungal that non-competitively inhibits the β-(1,3) d-glucan synthase by a different process than echinocandins, increased survival and reduced the fungal load in *C. auris*-infected mice treated for 21 days, in a dose-dependent manner, with the best response obtained at 25 mg/kg, resulting in the complete elimination of the fungus [106]. In vitro studies demonstrated that ibrexafungerp was more effective against *C. auris* than FLU, but inferior to isavuconazole and posaconazole [107]. Arendrup et al. [108] evaluated 122 clinical isolates of *C. auris* and confirmed its efficacy, with MICs ranging from 0.06 to 2 mg/L. Ghannoum et al. [109] tested ibrexafungerp in a guinea pig skin infection model and observed a significant reduction in the fungal load in the infected and treated tissues.

Rezafungin, an echinocandin with a prolonged half-life, low toxicity and intravenous administration, is a new candidate for the treatment of *C. auris* infections [110]. In vitro studies show that rezafungin MIC range was from 0.03 to 8 μg/mL, and the drug was effective even against isolates resistant to other echinocandins. In vivo in immunocompromised mice, rezafungin demonstrated its superiority compared to AmB and MICA, even with a lower frequency of administration [111].

Nikkomycin Z acts as a competitive inhibitor of chitin synthase, which is essential for the fungal cell wall. In vitro studies conducted by Bentz et al. [112], showed MIC values between 0.125 and >64 mg/L against *C. auris*, with resistance in Clade III. As there are no established cut-off points for resistance to nikkomycin Z, more studies are needed to understand its mechanisms and evaluate possible combinations with other antifungals. Another important study reported a synergistic effect between treatment with anidulafungin with nikkomycin Z or MICA with nikkomicin Z against *C. auris* isolates, obtaining significantly higher mortality rates when compared to isolated echinocandins [113].

Tu et al. [114] developed compound A1, based on the structure of NMU-6, which is a compound with a quinoxalinamide scaffold, an allyloxybenzene terminal group, and an oxyacetamide linker, that has an inhibitory action on proteins anchored to the fungal membrane. Compound A1 is of synthetic origin and was developed from phenotypic screening and structural optimizations. In vitro tests showed MICs between 0.06 and 2.0 μg/mL, while in vivo studies showed a significant reduction in renal fungal load in mice treated with 20 mg/kg.

Tetz et al. [115] evaluated the antifungal activity of the novel synthetic compound MYC-053 {*sodium5-[1-(3,5-dichloro-2-hydroxyphenyl) methylideneamino]-6-methyl-1,2,3,4-tetrahydro-2,4-pyrimidinedionate*} against five strains of *C. auris*. Its mechanism of action is still under investigation; however, it is believed that MYC-053 inhibits the chitin synthase of *C. auris*, which in turn mediates the synthesis of chitin from the fungal cell wall and suppresses the synthesis of nucleic acids inducing cell death. MYC-053 resulted in MICs of 1 to 4 μg/mL against *C. auris*.

As noted above, De Oliveira et al. [91] utilized the Pandemic Response Box to identify a compound active against *C. neoformans*, MMV1593537. This compound was also tested against *C. auris* resulting in MICs and MFCs (Minimum Fungicidal Concentration) of 5 μM for the strains tested.

### 3.4. Other Molecules and Vaccines

Singh et al. [67] developed a vaccine constituted with the N-terminal region of the Als3 proteins of *C. albicans* (NDV-3A). In a *C. auris* murine infection model, NDV-3A conferred 40% protection and fungal load in the kidneys and brain were lower compared to the non-immunized control. Protection could be enhanced by the administration of an antifungal as neutropenic mice, initially immunized with NDV-3A followed by infection with *C. auris* and then treated with MICA, had a >70% survival rate while the mice immunized with NDV-3A repeated the result of 40% and only 29% of mice receiving only MICA survived.

Di Mambro et al. [116] developed the first humanized monoclonal antibody against β-1,3 glucan. In vitro, studies evaluated the specificity of the antibodies and their fungicidal activity against *C. auris*. Flow cytometry and immunofluorescence analyses showed that H5K1 bound β-1,3-glucans on the surface of *C. auris*. An inhibition assay identified that the 250 and 100 µg/mL concentrations of H5K1 inhibited approximately 70% of growth, while the 50 µg/mL concentration inhibited 60%, the reduction in fungal adhesion on mammalian cells was 51.5% compared to the untreated control. The activity of H5K1 combined with CAS or AmB was also assessed and H5K1 + CAS together resulted in an MIC_50_ 0.125 µg/mL for CAS, while doses of 0.25–250 µg/mL of H5K1 were effective. Table 2 summarizes all studies evaluated in this work against *C. auris*.

## 4. *Aspergillus fumigatus*

### 4.1. New Treatment Options: Nanomedicine and Drug Combinations

Myriocin is an inhibitor of de novo sphingolipid synthesis by inhibiting the enzyme serine palmitoyl transferase (SPT). Myriocin reduced inflammation in a murine model of cystic fibrosis, indicating its potential role in lung diseases. To investigate whether nanoencapsulated myriocin could function as an antifungal, the drug was incorporated into solid lipid nanoparticles (SLNs) and these particles demonstrated good penetration into *A. fumigatus* biofilm. After 24 h of drug exposure, some hyphal forms underwent cell-wall collapse, and, after 48 h, the intracellular space was empty in most fungal cells. Thus, the incorporation of myriocin into a nanocarrier resulted in a fungistatic effect to *A. fumigatus*, leading to cell wall and biofilm impairment. Further studies are still needed to confirm its efficacy in vivo and safety in mammalian cells [117].

Nanoparticulated hydrogels, called nanogels, have good biocompatibility, controlled drug release and the possibility of carrying hydrophobic and amphiphilic drugs. The poly(glycidol)-based nanogel (PGBN) encapsulated with ITRA resulted in reduced MIC for *A. fumigatus*, from 0.19 µg/mL to 0.09 µg/mL. The novel nanogel also impaired biofilm activity. Although this nanogel did not reduce ITRA toxicity in mammalian cells, the reduction in MIC values may allow for lower concentrations of ITRA administration [118]

Domiphen is a cationic agent with biofilm disruption ability. Due to its ability to adsorb in the fungal surface and to penetrate in mannoproteins and chitin layers, this compound was tested in combination with ITRA. In a *Galleria mellonella* Aspergillus infection model, the synergy between the drugs led to decreased mortality and reduced signs of infection, such as reduced larval melanization. On the other hand, acute toxicity and cytotoxicity occurred with the concentrations of domiphen tested, particularly when compared to ITRA, further experiments are needed to clarify the safety of domiphen in treatment doses [119].

Triclosan is an antibacterial and antifungal agent used in toothpaste, mouthwashes, and cosmetics. Its mechanism of action relies on destroying biofilms through amino acids and fatty acids biosynthesis blockade and, thus, is a promising agent for antifungal purposes. The concomitant in vitro administration of triclosan and liposomal AmB (L-AmB) resulted in a synergic effect against *A. fumigatus*. At doses of 30% or 20% triclosan with L-AmB at its MIC, fungal viability was markedly reduced. Furthermore, the combinations impaired biofilm formation compared to L-AmB treatment only. The synergic effect was also observed by measuring reductions in *A. fumigatus* sph3 and ags3 gene expression. These genes are associated with galactosaminogalactan and α-1,3-glucan synthesis, respectively, and the latter facilitates conidia aggregation before biofilm formation. Combined therapy of triclosan and L-AmB may lead to reduced antifungal doses and improved results, with promising applications in fungal infections [120].

### 4.2. Drugs in Use or Under Investigation

ASP9726 is an echinocandin developed by Astellas Pharmaceuticals and published in 2014. In a guinea pig Aspergillus model, ASP9726 demonstrated a protective effect on animal survival at low doses (2.5 and 5 mg/kg). Although no reduction in lung fungal burden was observed, there were lower beta-glucan levels in sera from treated animals, with an overall reduction from ~1000 pg/mL to ~250–500 pg/mL. This indicates that ASP9726 had a beneficial action against aspergillosis [121].

The search for antifungal drugs targeting the CPY51A enzyme was first carried out in silico by Kritsi et al. [122]. These compounds were selected according to azoles’ physicochemical properties and binding interaction studies with a CYP51A model. Eight compounds were selected and purchased for MIC and minimum fungicidal concentration (MFC) evaluation. Although all compounds had antifungal activity, three were highlighted as the most promising ones—compounds 1, 2 and 4. Compounds 1 and 2 demonstrated the most potent antifungal effect against an *A. fumigatus* commercial strain (MICs of 0.1012 and 0.0507 µmol/mL, respectively), whereas Compound 4 had the lowest MIC for *A. fumigatus* clinical strain (0.1190 µmol/mL). However, these novel compounds were less potent than the azoles used as controls, econazole and ketoconazole.

Enfumafungin is a new class of antifungal drug related to inhibiting beta-1,3-glucan synthase, although it acts as a distinct target when compared to echinocandins. This compound is produced by the fungus *Hormonema carpetanum* and is currently used to develop derivative antifungal compounds [123]. To improve its stability, Apgar et al. [124] developed semi-synthetic structures derived from enfumafungin. The C3 aminoether derivatives obtained had good oral administration efficacy, without losing the enfumafungin property. Seven compounds were developed, with distinct substitutions, and one was selected as the most promising one, according to the minimum effective concentrations (MECs) obtained for *A. fumigatus* and MICs obtained for *C. albicans*.

Apgar and coworkers [106] examined the efficacy of ibrexafungerp in a murine aspergillosis model. The drug demonstrated a 90% increase in mice survival when compared to untreated mice, and the drug had good oral bioavailability. Furthermore, the drug showed a MEC of 0.25 μg/mL for *A. fumigatus*. Concurrent experiments with *C. albicans* resulted in determinations of an MIC of 0.06 μg/mL.

Drug repurposing is an interesting alternative to bypass the lack of new available fungal treatments since it relies on testing and validating the antifungal activity of already-approved drugs. In this regard, Auranofin, an FDA-approved drug for rheumatic disease treatment, gained attention due to its antibacterial and antiparasitic activity. Auranofin leads to cell death causing the release of reactive oxygen species (ROS) and was tested against a range of pathogenic fungi [125]. Wiederhold et al. [125] identified auranofin as a potential drug for *A. fumigatus*, with good MIC values against *A. fumigatus* (2–4 μg/mL), *Scedosporium apiospermum* (1–4 μg/mL) and *Lomentospora prolificans* (2–8 μg/mL). Chen et al. [126] tested the compound against a larger panel of *A. fumigatus* clinical strains and demonstrated that auranofin was also efficient against ITRA- resistant strains, impairing biofilm formation and maturation. Furthermore, the compound showed a synergistic effect with ITRA and AmB even against ITRA-resistant strains, reducing the MIC for ITRA and AmB when concomitantly administered with auranofin.

A new class of antifungal drugs, the orotomides, gained attention for distinct fungal pathogens. Its main drug, olorofim (F901318), a new antifungal drug belonging to the class of orthonyms that acts by impairing the pyrimidine biosynthesis pathway [127]. This fungal pathway is relevant for a range of processes, from genetic-material synthesis to cell-wall synthesis [128]. At present, olorofilm has an FDA Breakthrough Therapy and Orphan Drug Designation for the treatment of invasive mold infections in patients with few (or no) treatment options as well as for coccidioidomycosis of the central nervous system refractory to standard-of-care antifungal therapy [127]. Du Pré et al. [128] found that olorofim impaired the germination of *A. fumigatus* during the first hours after exposure, but the fungus maintained its isotropic growth. Furthermore, prolonged exposure reduced polarized growth of the hyphal form and damaged cell-wall deposition. The authors concluded that olorofim had a fungistatic effect on conidia, while it produced a fungicidal effect after 10 h of hyphal exposure. Hence, the drug is effective mainly against hyphal morphotype, in a time dependent manner.

Manogepix (MGX), the active metabolite of fosmanogepix, effectively reduced lung fungal burden in mice infected with *A. fumigatus* compared to control animals, including when azole-resistant and echinocandin-resistant isolates were evaluated, in a concentration-dependent manner [82]. In *A. fumigatus* azole-resistant strains, MECs ranged from 0.016 to 0.125 mg/L [129]. In a larger collection of global *A. fumigatus* isolates, MECs varied from 0.008 to 0.06 mg/L [130].

Other candidates with potential antifungal activity to *A. fumigatus* that are in clinical development include opelconazole (the only antifungal tested for inhalation administration) and BAL2062 (a first-in-class siderophore antifungal) [131]. Opelconazole, a novel triazole with similar action to azoles, is targeted for the treatment of pulmonary aspergillosis via inhalation administration through nebulization [127]. Following intravenous treatment in neutropenic mice infected with *A. fumigatus*, opelconazole showed potential action against the pathogen with significant inhibition of the fungal load [132].

Similar to its efficacy against *C. auris*, turbinmycin has potent antifungal activity against *A. fumigatus*. Turbinmycin significantly reduced *A. fumigatus* burdens in the lungs of infected mice. Notably, safety was also demonstrated, as the mice appeared healthy after 16 administrations of turbinmycin during the 4-day treatment period [105].

### 4.3. Natural Products

The activity of cinnamaldehyde was compared to VORI in an immunosuppressed murine *A. fumigatus* infection model. The results demonstrated a greater reduction in lung fungal burden (80%) and increased cure rate (80%) in mice treated with cinnamaldehyde for 14 days. The reduction in fungal load and pathological efficacy in the group treated with VORI was 67.70% and 66.70%, respectively. Additionally, electron microscopy demonstrated thinner hyphae cell walls in the lungs of mice treated with cinnamaldehyde compared to those treated with VORI. Many hyphae lost their cell walls, presumably due to the drug effect on beta-1,3-glucan synthesis, as cinnamaldehyde is absorbed in the cell wall, breaking the polysaccharides, reaching the beta-1,3-glucan synthesis pathway, and impairing fungal growth and reproduction [133].

*Myristica fragrans* is used in meal preparation as a condiment. Its extracts contain chemical compounds such as alkyl benzene derivatives, beta-pinzene, and terpenes, and this spice has known medicinal antidepressant and antioxidant properties, among other actions [134]. Extracts from *M. fragrans* were obtained using distinct solvents—chloroform, methanol, ethanol, and hexane—and were evaluated for their MECs against *A. fumigatus*. The most effective MEC (0.078 mg/mL) for *A. fumigatus* was from the extract obtained with hexane, and further analyses were carried out with this solution. The hexane extract reduced ergosterol concentration in the *A. fumigatus* membrane, which was similar to that achieved with AmB. Further, there was a 76.09% reduction in cell wall melanin and impairments in cell wall hardness. Another product of *M. fragrans* is isoeugenol, an essential oil that is also produced by *Cinnamomum verum*, and the oil has known antioxidant and antibacterial properties. The analysis of isoeugenol activity against *A. fumigatus* revealed a reduction in rodlet layer hydrophobicity and a downregulation of the RodA gene. RodA is the main protein responsible for the hydrophobic characteristic of *Aspergillus* conidia [135].

Hoda et al. [135] also evaluated the compound cis-9-hexadecenal, an organic aldehyde with antibacterial and anti-inflammatory characteristics found in *M. fragrans* and some other plants. Cis-9-hexadecenal reduced *A. fumigatus* (conidial) hydrophobicity, from almost 90% to less than 60%, and melanization was reduced by more than three times, with direct interference in the melanin biosynthesis pathway. Isoeugenol, a phenylpropanoid found in essential oils, also impaired *A. fumitatus* melanin formation through gene downregulation. Biofilm formation was also reduced in the presence of this compound [136]. Table 3 summarizes all studies evaluated in this work against *A. fumigatus*

## 5. *Candida albicans*

### 5.1. New Treatment Options: Nanomedicine and Drug Combinations

Khan et al. [137] developed a cinnamaldehyde-encapsulated multilamellar liposome, and the new liposome was rich in vacuolar fragmentation and disintegration, as well as loss of cell-wall integrity. In addition, the Hwp1 gene, involved in hyphae formation, was upregulated in terms of quality after exposure to the cinnamaldehyde-encapsulated liposome and, according to the results, was downregulated. Liposomes were also used to encapsulate VORI. The VORI liposomes interacted with the chitin present in the cell wall of *C. albicans*, improving the antifungal potential of VORI [138].

Salehi et al. [139] evaluated the potential of gold nanoparticles to improve the efficacy of loaded CAS, and these particles increased the efficacy of CAS. The MIC results showed that the nanoparticle significantly decreased the concentration. Among all isolates, a MIC ≥4 µg/mL was observed for CAS, except for *C. glabrata*. However, the MIC50 of CAS conjugated to gold nanoparticles against *C. albicans* was 0.03 μg/mL. Microscopy data showed that the cell-wall structure was altered by the effect of loaded CAS. Marena et al. [96] evaluated MICA loaded in a nanoemulsion against *C. albicans* (and other species). The nanoemulsion intensified the antifungal activity of MICA against mature biofilms. For example, at a concentration of 1.56 µg/mL, the reduction in metabolic activity of mature *C. albicans* biofilm was 68.3% and 52.4% for nanoemulsion encapsulated with MICA and free MICA, respectively.

### 5.2. Natural Products

5,11-Dimethyl-5*H*-indolo[2,3-b]quinoline (DiMIQ) is a synthetic analog of neocryptolepine, a minor alkaloid from Cryptolepis sanguinolenta. Zarnowski et al. [140]. evaluated the antifungal activity of DiMIQ against *C. albicans* and found that the treatment caused significant changes in the production of the fungal cell wall. The composition of the wall was altered when compared to the control group. For example, the level of hexoses, which are important in the construction of carbohydrate polymers, glucans and mannans, was increased by 4.8% to a total of 92.2%. Furthermore, the treatment caused a detrimental effect on the content of pentoses (ribose, xylose and arabinose) in *C. albicans* biofilms.

Aucklandia lappa Decne or Saussurea lappa, popularly known as costus or kuth, was evaluated against *C. albicans* by Lee et al. [141] and exposure to the compound increased the intensity of Calcofluor White, demonstrating a change in the cell wall. After 3, 5 and 6 h of incubation with the natural product, the inhibition of chitin was 92.1%, 84.6% and 79.8%, respectively. Glucans were inhibited by 84.3%, 79.7% and 70.2% after treatment with a final concentration equal to its MIC, 2× MIC and 4× MIC.

LL-37 is the only member of the human cathelicidin family of antimicrobial peptides (AMPs) and its potential mechanism of action against the cell wall of *C. albicans* was investigated in detail by Hsu et al. [142]. Treatment with LL-37 resulted in cell-wall stress, induction of reactive oxygen species, activation of the endoplasmic reticulum-related unfolded protein response and altered protein secretion. These changes may be related to the mechanism of action of LL-37, acting on cell-wall components, remodeling the wall and reducing cell adhesion. The authors also analyzed a possible relationship between LL-37 and Sfp1, a C2H2-type zinc finger transcription factor, and showed that Sfp1 contributes to the regulation of stress responses. Two studies of Tsai et al. [143,144], reported that LL-37 activity may interact with the cell wall of *C. albicans* by interacting with polysaccharides, especially exoglucanase Xog1 and mannans. LL-37 causes wall remodeling and exposure to β-glucan in *C. albicans*. These effects result in a decrease in the ability of *C. albicans* to adhere to surfaces.

Cannabidiol is a product derived from cannabis sativa and its antifungal profile was evaluated by Feldman et al. [145]. Treatment with cannabidiol caused significant changes in the cell wall of *C. albicans*. A dose-dependent reduction in chitin was observed. At concentrations of 12.5, 25, and 50 μg/mL, there was a reduction of 37%, 53%, and 90% in chitin, respectively. Pseudohyphae with finer septations occurred after treatment with cannabidiol. Thus, with the reduction in chitin, the morphology of the yeast was notably altered.

Phloretin is a dihydrochalcone flavonoid present in apples and strawberries. Liu et al. [146] observed that this compound displayed anti-*C. albicans* activity, with significant inhibition in the formation of pseudohyphae by more than 70% at concentrations of 1× or 2× MIC. Treatment with phloretin decreased the expression of the genes associated with cell elongation extension 1 (ECE1), Hwp1, enhanced adhesion to polystyrene 1 (EAP1) and agglutinin-like sequence 3 (ALS3) in a dose-dependent manner. Thus, phloretin suppresses the transition of yeasts into pseudohyphae, limiting their virulence and pathogenicity.

Microbial metabolic extracts, peptides and bacteriocins, for example, are being investigated as medicines and preservatives. Wang et al. [147] investigated the activity of an enterocin isolated from Enterococcus faecalis TG2 (CHQS) against *C. albicans*. Enterocin treatment increased the β-1,3-glucan of *C. albicans* composition compared to untreated cells, increasing as much as 3.4-fold after 256 μg/mL of CHQS. Similarly, chitin composition increased 2.9-, 2.2-, and 2-fold at 64, 128, and 256 μg/mL CHQS, respectively. Microscopically, cell-wall ruptures (white cavitations) and intracellular vacuolization were also reported.

Fleagrass (Adenosma buchneroides Bonati) is a natural product widely used as an insect repellent and its antifungal activity was evaluated by Wu et al. [148]. Fleabane and carvacrol disrupted *C. albicans* cell wall structure, exposing glucans. Regarding chitin, the calcofluor fluorescence intensity was noticeable for the group treated with fleabane and carvacrol, with greater intensities achieved following treatment with fleabane.

Jinyi Liu et al. [149]. investigated the antifungal profile of a β-carboline alkaloid methylaervine (MET) against *C. albicans*. The MICs of MET ranged from 16 to 128 μg/mL for *C. albicans*. Electron microscopy analysis revealed that MET damaged the cellular structure, including the cell wall, among other structures.

### 5.3. New Antifungal Drugs

Quindós et al. [150], evaluated the profile of ibrexafungerp against different *Candida* species and found that MICs ranged from 0.016 to ≥8 mg/L, with the lowest MIC being for *C. albicans* (0.062 mg/L, MIC range 0.016–0.5 mg/L). These results corroborate other studies, such as in Schell et al. where the drug displayed MICs ranging from 0.06 to 0.25 µg/mL. Mesquida et al. [151] reported MICs from 0.03 mg/L to 0.25 mg/L against *C. albicans*.

Enfumafungin, described above, was evaluated by Jiménez-Ortigosa et al. [152]. The drug displayed an average MIC of ≤0.03 µg/mL (≤0.03–0.06 µg/mL) against *C. albicans* without serum and an MIC of 0.5 µg/mL (0.5–1 µg/mL) against *C. albicans* in medium with 50% serum.

Arendrup et al. [153] observed that rezafungin showed efficient antifungal activity against Candida species, and documented low MICs against *C. albicans* (0.016, 0.002–0.125 mg/L). In Locke et al. [154], the MIC value ranged from 0.03 to 0.25, depending on the *C. albicans* strain (in RPMI medium). The values in the studies varied depending on the culture medium used. Time-kill experiments showed that rezafungin behaved in a dose-dependent fungicide manner in most of the assessed concentrations (2–128 µg/mL). Using 125 *C. albicans* isolates, Tóth et al. [155] obtained a MIC90 of 0.06 mg/L. An early access study performed in Italy and Germany, demonstrated that patients with cadidemia who received rezafungin for up to 39 days as outpatient therapy had a better quality of life, and the drug was generally effective and well tolerated [156]. The rezafungin STRIVE study showed a significant reduction in ICU length of stay for the group of patients who received rezafungin treatment, contributing not only to patient recovery but also to economic benefits [157]. The phase 3 ReSTORE study with 246 patients with candidemia that either received treatment with rezafungin (122 patients) or caspofungin (124 patients) confirmed the overall efficacy and safety of rezafungin, with early efficacy related to concentrated initial exposure [158].

Zhao et al. [159] demonstrated that fosmanogepix in combination with 1-aminobenzotriazole decreased the fungal load in *C. albicans* infected mice. The 1-aminobenzotriazole increased plasma exposure to fosmanogepix in mice, increasing its efficacy. Also, fosmanogepix (26 mg/kg) alone moderately reduced the fungal load (0.2 log10 CFU/g).

Notably, in 2022, oteseconazole, administered orally, became the first FDA approved drug for recurrent vulvovaginal candidiasis (RVVC) in the United States of America [160] (n.b. ibrexafungerp was subsequently approved for RVVC later in the same year, though it was approved for general vulvovaginal candidiasis in 2021). In a clinical trial of RVVC, oteseconazole was statistically more effective than fluconazole, achieving a therapeutic cure rate of 66.88% compared to 45.91% with fluconazole. Furthermore, mycological and clinical cure rates with oteseconazole were 82.50% and 71.25%, respectively, compared to 59.12% and 55.97% with fluconazole, respectively [161].

### 5.4. Other Molecules and Vaccines

The combination of sodium nitroprusside, an antihypertensive, and the alternative oxidase inhibitor salicylhydroxamic acid (SNP-SHAM) resulted in significant defects in *C. albicans* cell wall organization, leading to fungal unmasking and immune recognition in cell culture and animal models [162]. Notably, the masking of cell wall components facilitates evasion of the immune system [163]. Duvenage et al. [162] observed that this combined treatment caused the loss of viability and rearrangements of the fungal cell wall, increasing the rate of macrophage uptake. Staining with Calcofluor-white and Congo red revealed a reduced thickness in the outer cell wall, but not in the inner wall. Despite an increased exposure of chitin and β-glucans, SNP+SHAM had no significant effect on chitin, glucan and mannans. Hence, SNP+SHAM may cause changes in the organization of the wall and not in its components.

Nicotinamide, an amide form of vitamin B3, has been extensively investigated for its antimicrobial potential. Xing et al. [164], for example, observed that nicotine treatment increased the fluorescence signal at the periphery of *C. albicans*’ cell wall, indicating an increased exposure of β-glucans on the surface of the outer cell wall. Furthermore, nicotinamide caused a decrease in mannans compared to untreated cells. Also, the Calcofluor-white method revealed an increase in chitin after treatment. In addition to these changes in morphology, the authors found that nicotinamide caused an enlargement of the cells and induced the formation of pseudohyphae.

The antifungal profile of cyclohexylidene-4-phenyl-thiazole was investigated by Nívea et al. [165]. Cyclohexylidene-4-phenyl-thiazole caused a decrease in the ability of *C. albicans* to adhere to epithelial cells of the oral cavity and prolonged the survival of infected *C. elegans* parasites. Furthermore, the presence of a thinner cell wall was observed after treatment, especially the layer composed of glucans. Treatment reduced the amount of 1,3-β-d-glucan in the cells.

Wang et al. [166] used hybridoma technology to develop a C12 and C346 monoclonal antibody directed against recombinant *C. albicans* mannosyltransferase 4 (rPmt4p) protein. Antibody treatment reduced yeast burden as well as in the inflammatory process in the kidneys of mice infected with *C. albicans*. Furthermore, the antibodies activated macrophage-mediated opsonophagocytosis and neutrophil killing. Table 4 summarizes all studies evaluated in this work against *C. albicans*.

## 6. Conclusions

The studies discussed in this review article evaluated the antifungal profile of different promising agents, including natural products, recently approved drugs, new molecules, antibodies, antigens, and even new nanocarriers. We acknowledge that there are other products in pre-clinical development against these WHO critical threat pathogens and apologize to those investigators whose work we did not highlight, but the representative therapeutics described exemplify the ongoing efforts of the field. All the studies discussed point to a positive outlook for the development of new treatment options, expanding the arsenal of antifungals and contributing to the control of infectious diseases, especially the fungal diseases considered a critical priority by the WHO. The studies also highlight the reality of the many challenges related to the discovery of new molecules with antifungal potential. The low interest in developing new molecules and the worrying neglect regarding fungal diseases remain a major concern. Furthermore, the increase in new isolated species (and the expectation for additional emerging pathogens) and the low susceptibility to drugs are additional challenging factors facing medical mycology. Nevertheless, there is still hope for a promising future as there is a slow increase in the awareness of the urgent need for improved control of fungal diseases. It remains essential to emphasize that the road ahead is long and complex, but given the work detailed in this review, it is expected that new therapies will become a reality soon. Meanwhile, the number of patient deaths from fungal infections is currently increasing every day.

## Figures and Tables

**Table 1:** Summary of in vitro and in vivo studies with different promising anti-cell wall therapies involving *Cryptococcus neoformans*.

Target	Sample	Methods Used	Activity (Result Against Fungus)	Ref.
Cell wall and capsule	Silver nanoparticles (SNPs) from the fungus *Fusarium oxysporum*	Production and characterization of the SNP; antifungal activity assay; scanning electron microscopy	Cell wall disruptions and increased thickness; cytoplasmic membrane alterations and loss of the inside content	[79]
Calcium channel inhibition	Verapamil (VER)	Quantification of cell surface molecules; fluorescence microscopy; calcium measurement; stresses susceptibility assay; in vivo assay on *G. mellonella* model	Membrane permeability alteration; chitosan reduction; cell wall disruption; melanin leakage; sensitivity to Caspofungin	[80]
Gw1t enzyme	APX001A and its prodrug, APX001	Antifungal activity assay; in vivo assay on murine model; fungal burden analysis; pharmacokinetic analysis	Mislocalization of glycosylphosphatidylinositol (GPI)-anchored mannoproteins	[81]
F-actin cytoskeleton	LA (Lipoic Acid)	Phase-contrast and fluorescent microscopy, flow cytometry, and transmission electron microscopy	Arrested cellular proliferation; ultrastructural disorder; aberrant cell walls.	[83]
Ergosterol biosynthesis and capsule	Essential oil of *Ocimum basilicum* var. Maria Bonita	Antifungal activity assay; ergosterol quantification assay; electron microscopy; cytotoxicity assay	Capsule reduction and disruption; membrane irregularities and invaginations; cell wall thickening	[85]
Cell wall and capsule	α-Cyperone from Cyperus rotundus’s essential oil	Antifungal activity assay; PI uptake assay; India ink assay; checkerboard assay	Reduction in capsule thickness; acapsular cells; easily broken cells	[86]
Mannoproteins, glucuronoxylomannan and galactoxylomannan	Ubiquicidin (UBI) and its fragments	Cytotoxicity assay; antifungal activity assay; peptide synthesis mass spectrum; high performance liquid chromatography purification	Cell surface electrostatic imbalance; growth inhibition	[87]
Cell Wall Integrity and Pigmentation pathway (CWIP) pathway	Benzothioureas	NIH Molecular Libraries Initiative screening; antifungal activity assay; adenylate kinase assay; cytotoxicity assay; Western blotting; cellular protein kinase activity	Inhibition of Mpk1 phosphorylation and CWIP pathway inhibition; cell wall impairment	[88]
Cell wall and membrane	MMV688271	The Pathogen Box—MMV screening; antifungal activity assay; cell viability assay; electronic microscopy; sorbitol assay	Fungicide effect; aberrant cell wall and membrane; tightly packed organelles	[89]
Glucuronoxylomannan	MSI-1 (Antimicrobial peptide)	Zeta potential analysis; flow cytometry; antifungal activity assay; both transmission electron and fluorescence microscopy; in vivo assay on murine model	Increase in membrane fluidity and permeability leading to its integrity loss	[90]
Chitinase-mediated hydrolysis of chitin	MMV1593537	Pandemic Response Box screening; antifungal activity assay; cytotoxicity assay; antifungal characterization; electron microscopy	Repression of capsule formation; capsule thickness reduction; increase in chitooligomers	[91]
Cell membrane viability and stability	EIPE-1	Cytotoxicity assay; antifungal activity assay; electron microscopy; RNA analysis; in vivo assay on *G. mellonella* model	Aberrant cell structures; capsule and ergosterol biosynthesis impairment; cell wall and membranes remodeling; internal content leak	[93]
Stimulation of the host immune response	Capsule as an adjuvant to a CDA1-LNP vaccine	Synthesis of mRNAs encoding the antigen; synthesis and characterization (NanoFCM INC) of lipid nanoparticles; in vivo assay on murine model; Western blot, capitule purification; mCherry assay	Reduced fungal burden	[94]

**Table 2:** Summary of in vitro and in vivo studies with different promising anti-cell-wall therapies involving *Candidozyma auris*.

Target	Sample	Methods Used	Activity (Result Against Fungus)	Ref.
1,3-β-d-glucan	Nanoemulsion co-encapsulated with amphotericin B and micafungin	In vivo study in leukopenic mice. The fungal load of the lung, thymus, kidneys, spleen and liver was analyzed.	The co-encapsulated nanoemulsion significantly reduced the fungal load in the tissues in a shorter treatment time when compared to the free or nanoencapsulated drugs separately	[95]
1,3-β-d-glucan	nanoemulsion of micafungin	In vitro: Inhibition of biofilm formation. In vivo: In a *Galleria mellonella* model with quantification of the fungal load.	NEM had its most effective activity on mature biofilms, and in the in vivo study the nanoemulsion potentiated the activity of micafungin, resulting in a reduction in the fungal load compared to the controls.	[96]
1,3-β-d-glucan and chitin	Traditional Herbal Monomers	MIC	The MIC of the Traditional Herbal Monomers (THMs) separately ranged from 50 to 64 mg/mL. While the best combination was CIN/PAL with an MIC of 12.5 mg/mL. All the compounds were effective in combating *C. auris*.	[97]
Cell wall	*Cinnamomum zeylanicum Blume* bark essential oil and *C. zeylanicum Blume* leaf essential oil	MIC and MFC	The MIC and CMFs of CBEO were lower than 0.03% (*v*/*v*), while CLEO showed a result of 0.06% (*v*/*v*) and 0.25% (*v*/*v*) for MIC and CMFs, respectively, so CBEO showed better antifungal activity.	[98]
Cell membrane	alkaloids solenopsins from fire ants (natural fire ant extract or a synthetic mixture of analogs)	MIC, Synergism, acute toxicity assay in *G. mellonella*, cytotoxicity assay and systemic infection model in *G. mellonella*	IC50: 1.4–0.7 µg/mL for natural fire ant extract and synthetic mixture of analogs FIC (AmB + alkaloids): 0.59–1.03 for Checkerboard test and 1.58–3.03 for Bliss model Significant reduction in biofilm mass after treatment with alkaloids (except at a concentration of 10 µg/mL). Treatment with a natural fire ant extract or a synthetic mixture of analogs significantly reduced the metabolic activity of mature biofilms. Rupture of the fungal cell membrane Increased survival rate of larvae infected and treated with alkaloids.	[100]
*lysine acetyltransferase*	CPTH2 and caspofungin	Fungal growth (Absorbance 600 nm)	Lower growth of *C. auris* was obtained in the culture with the presence of CPTH2 + CAS with results below 0.5 in absorbance, while the monotherapies the growth was approximately 1.0 in absorbance.	[101]
Fungal protein Gwt1	Fosmanogepix	In vitro: MIC by broth microdilution according to CLSI M27-A3 guidelines. In vivo: Fungal load in kidney, lung and brain tissue per CFU/mL	Anidulafungin and Fosmanogepix were effective in reducing the fungal load in the kidneys and lungs, but only Fosmanogepix reduced the fungal load in the brain.	[102]
Fungal protein Gwt1	Fosmanogepix	MIC by broth microdilution according to CLSI guidelines	MIC of fosmanogepix ranged from <0.005 to 0.015 mg/L, with a mean of <0.005 mg/L, MIC_50_ and MIC_90_ were 0.002 and 0.008 mg/L respectively, and there was no significant difference between clades and resistant isolates.	[103]
Fungal protein Gwt1	Fosmanogepix	In vitro broth microdilution test according to EUCAST and CLSI. Phase 2 study in humans.	MIC using the CLSI (0.008 to 0.015 μg/mL) and EUCAST (0.004 to 0.03 μg/mL) test methods. All patients had negative blood cultures at the end of treatment.	[104]
Yeast phosphatidylinositol transfer protein (Sec 14p)	Turbinmycin	-	Important action against yeast phosphatidylinositol transfer protein (Sec 14p). Mice infected with *C. auris* were treated with turbinmycin with a logarithmic reduction of 3.610	[105]
1,3-β-d-glucan	Ibrexafungerp	MIC_90_ and growth inhibition evaluation	Ibrexafungerp inhibits the growth of the fungus due to the morphological changes it causes to the pathogen and the MIC_90_ was 1 mg/L. The drug is therefore effective.	[107]
1,3-β-d-glucan	Ibrexafungerp	MIC Evaluation for Antifungal Effectiveness	Ibrexafungerp was active against all clinical isolates, with MICs ranging from 0.06 to 2 mg/L with an average of 0.5 mg/L.	[108]
1,3-β-d-glucan	Ibrexafungerp	CFU/mL of skin tissue	The antifungal activity was successful, with a reduced fungal load with Ibrexafungerp treatment (no quantification data).	[109]
1,3-β-d-glucan	Rezafungin	MIC_50_ and MIC_90_	The MIC of the different clades showed no statistically significant difference. The MIC ranged from 0.03 to 8 μg/mL, while the MIC_50_ and MIC_90_ were 0.125 μg/mL and 0.5 μg/mL, respectively.	[110]
1,3-β-d-glucan	Rezafungin	CFU/mL in renal tissue	Rezafungin showed a greater reduction in the fungal load in kidney tissue than amphotericin B and micafungin. The highlight of rezafungin is the lower number of doses over the course of treatment.	[111]
*Chitin Synthase Enzyme*	Nikkomycin Z	MIC by broth microdilution as determined by CLSI	The results obtained among the different clades were similar, with the exception of Clade III. The MIC results in this clade ranged from 16 to >64 mg/L and MIC_50_ was 32 mg/L, while the average MIC of the other clades ranged from 0.125 to >64 mg/L, with an overall average of 2 mg/L, MIC_50_ and MIC_90_ were 2 and 32 mg/L, respectively.	[112]
*Chitin Synthase Enzyme*	Echinocandins + Nikkomycin Z	MIC and Time Kill	MIC of anidulafungin, micafungin, and nikkomycin Z: 0.015–4, 0.03–4, and 2–>16 mg/L, respectively. Anidulafungin and micafungin isolates showed low antifungal activity against wild-type isolates and the isolate with a mutation in the FKS1 hotspot region 2. However, they showed significant antifungal activity against isolates with a mutation in the hotspot region 1. A total of 36.7% of the strains were susceptible to anidulafungin combined with nikkomycin Z, and 40% were sensitive to micafungin with nikkomycin Z, with fungicidal effects of 41.7% and 20%, respectively, against wild-type isolates.	[113]
Inhibition of *Glycosylphosphatidylinositol* (GPI)	Compound A1	MIC, human cell toxicity and fungal load of infected and treated mouse liver tissue.	MICs among the different isolates ranged from 0.06 to 2.0 μg/mL. The toxicity in human cell lines was IC_50_ > 29 μg/mL, showing low toxicity. In vivo study, the renal fungal load was significantly lower in the animals treated with compound A1 compared to the untreated control.	[114]
Inhibition of chitin synthase	Compound MYC-053	MIC	MIC ranges from 1 to 4 µg/mL among the strains analyzed, demonstrating sensitivity to compound MYC-053.	[115]
Enzymatic hydrolysis of chitin	Compound MMV1593537	MIC, MFC and cytotoxicity in macrophages (RAW 264.7)	The MIC and CFM results were 5 μM for both strains. The cytotoxicity test showed that the compound had no significant toxicity	[91]
1,3-β-d-glucan	NDV-3A and Micafungin	Survival rate after challenge	The animals that received double therapy, immunization with NDV-3A and treatment with micafungin after infection had a survival rate of 70%, higher than the controls with only one therapy.	[67]
1,3-β-d-glucan and chitin	H5K1 monoclonal antibody	In vitro studies of growth inhibition, fungal adhesion to mammalian cells and MIC_50_	The 250 and 100 µg/mL concentrations of H5K1 inhibited approximately 70% of the growth of *C. auris*, while the 50 µg/mL concentration inhibited 60%, and the reduction in fungal adhesion to mammalian cells was 51.5%. The MIC evaluated the activity of H5K1 with caspofungin (CAS) and amphotericin B (AMB), the combination H5K1 + CAS was the one that showed the best result with MIC_50_ 0.125 µg/mL	[116]

CPTH2: *cyclopentanone, 2-[4-(4-chlorophenyl)-2-thiazolyl] hydrazone*; MIC: Minimum inhibitory concentration; MFC: Minimum fungicidal concentration; CFU: Colony.

**Table 3:** Summary of in vitro and in vivo studies with different promising anti-cell-wall therapies involving *Aspergillus fumigatus*.

Target	Sample	Methods Used	Activity (Result Against Fungus)	Ref.
Serine palmitoyl transferase enzyme	Af293 (Myriocin + SLNs)	In vitro broth microdilution, following EUCAST guideline for planktonic cells, to define MEC. In vitro XTT reduction assay for biofilm evaluation. CLSM for biofilm evaluation.	Impaired cell wall and biofilm formation, cell-wall collapse	[117]
Hyphae cell wall Ergosterol (ITZ mechanism)	(PGBN + ITZ)	In vitro broth microdilution for MIC determination, using the CLSI guideline. In vitro biofilm formation evaluation. In vitro cytotoxicity test analyzed through luminescent cell viability assay.	Reduced ITZ MIC	[118]
Chitin and mannoprotein interaction Ergosterol (ITZ mechanism)	(Domiphen + ITZ)	In vitro broth microdilution for MIC evaluation through CLSI guideline. In vitro biofilm and cell wall analysis. In vivo acute toxicity in zebrafish model. In vivo antifungal efficacy in *Galleria mellonella* larvae.	Reduced ITZ MIC; reduced mortality and melanization in larvae	[119]
Galactosaminogalactan and α-1,3-glucan biosynthesis	Triclosan + L-AMB	In vitro broth microdilution for MIC evaluation, following the EUCAST guideline. In vitro evaluation of triclosan and L-AMB in combination through fractional inhibitory concentration index (FICI). In vitro biofilm evaluation through crystal violet assay.	Synergistic effect, with fungal viability reduction	[120]
β-1,3-glucan	ASP9726	In vivo ASP9726 efficacy evaluation in guinea pig model (single and multiple doses, in 2.5, 5 and 10 mg/kg, subcutaneous administration). Treatment evaluation through pulmonary fungal burden (CFU/g), histopathology and β-d-glucan and galactomannan levels.	Reduced mortality and serum beta-glucan detection	[121]
CYP51A enzyme	Compounds 1, 2 and 4	In vitro microdilution broth, following the CLSI guideline, for MIC and MFC evaluation.	Higher MIC when compared to ITZ—possible structural changes to improve the compounds	[122]
β-1,3-glucan synthase enzyme	(Enfumafungin-derived structures through C2 replacement with heterocycles or C3 replacement with aminoethers)	In vitro broth microdilution, according to CLSI guideline, to define the MECs for *A. fumigatus* MF5668.	Some compounds presented low MICs	[124]
Extracellular matrix (exact mechanism of action still unknown)	Auranofin	In vitro microdilution broth, according to the CLSI guideline, to define the MIC50 and MIC100.	Fungal death through ROS release	[125]
Extracellular matrix (exact mechanism of action still unknown)	Auranofin and auranofin + ITZ or AMB	In vitro microdilution broth, following the CLSI guideline, for MIC evaluation. In vitro checkerboard assay for interaction evaluation between auranofin, ITZ and AMB. In vitro biofilm formation evaluated by crystal violet assay. and microscopy)	Low MICs alone; synergistic effect with ITZ and AMB, increasing susceptibility in resistant strains	[126]
Pyrimidine biosynthesis	Olorofim—F901318	In vitro analysis through transmission electron microscopy (TEM) for growth analysis, and confocal microscopy viability evaluation.	Impaired germination and hyphae cell wall disruption	[128]
GPI-anchor biosynthesis (Gwt1 protein)	AF293, AF41, EMFR S678P, F11628, AF72 and F14532 isolates (APX001A—MGX and APX001—FMGX)	In vitro broth microdilution, according to the CLSI guideline, to evaluate MEC results for APX001A. In vivo evaluation of APX001 treatment with fungal burden and dose–response evaluation.	Lower MICs when compared to ITZ, VRC and POS	[82]
GPI-anchor biosynthesis (Gwt1 protein)	MGX—APX001A	In vitro microdilution broth, according to the EUCAST guideline, to evaluate MGX MEC in comparison to other EUCAST- and CLSI-based studies.	Reduced MIC/MEC when compared to azoles; reduced fungal burden at the same level of azoles	[129]
Yeast phosphatidylinositol transfer protein (Sec 14p)	Turbinmycin		Important action against yeast phosphatidylinositol transfer protein (Sec 14p). Significant inhibition of fungal load in lungs after treatment with 1 mg/kg. Dose-dependent reductions in fungal load with a 1.5 logarithm.	[105]
GPI-anchor biosynthesis (Gwt1 protein)	MGX—APX001A	In vitro broth microdilution, according to the CLSI guideline, to evaluate MIC and MEC.	Lower MECs for itraconazole-resistant strains	[130]
lanosterol 14α-demethylase	Opelconazole	In vitro and in vivo in immunocompromised mice	Inhibition of pulmonary fungal load and reduction in galactomannan concentrations in bronchoalveolar lavage fluid and serum, as well as in several biomarkers in a dose-dependent manner, being >3 and >47 times more potent than other antifungals, such as posaconazole and voriconazole.	[132]
β-1,3-glucan biosynthesis	Cinnamaldehyde	In vivo evaluation of 14-day treatment with cinnamaldehyde, in comparison to voriconazole (both through oral administration). The used methods were microscopy, culture, histopathology and β-d-glucan dosage in the lung.	Lower MICs and MECs when compared to azoles	[133]
Ergosterol and melanin biosynthesis	*Myristica fragrans* hexane extract	In vitro broth microdilution test, according to the CLSI guideline, to identify the MEC of each compound. In vitro evaluation of fungal structure through TEM.	Reduced mortality and fungal burden; thinner hyphae cell wall	[134]
Melanin biosynthesis	*Cis*-9-hexadecenal	In vitro microdilution broth, according to the CLSI guideline, to define the compound’s MEC. In vitro melanin production evaluation. In vitro SEM and TEM microscopies analysis of the cell surface.	Reduced rodlet layer hydrophobicity and melanin formation	[135]
RodA and gene expression melanin biosynthesis	Isoeugenol	In vitro microdilution broth, following the CLSI guideline, to identify MIC100 and MIC50. In vitro TEM, SEM and CLSM evaluation of biofilm and conidia surfaces.	Low MEC, reduced melanin and ergosterol concentration	[136]

SLNs: solid lipid nanoparticles. MIC: minimum inhibitory concentration. MEC: minimum effective concentration. MFC: minimum fungicidal concentration. CFU: colony forming unit. XTT: 2,3-bis(2-methoxy-4-nitro-5-sulfophenyl)-2*H*-tetrazolium-5-carboxanilide inner salt. CLSM: confocal laser scanner microscopy. EUCAST: European Committee on Antimicrobial Susceptibility Testing. CLSI: Clinical and Laboratory Standards Institute. PGBN: poly(glycidol)-based nanogel. ITZ: itraconazole. L-AMB: liposomal amphotericin B. FICI: fractional inhibitory concentration index. TEM: transmission electron microscopy. SEM: scan electron microscopy. FMGX: fosfomanogepix. MGX: manogepix.

**Table 4:** Summary of in vitro and in vivo studies with different promising anti-cell-wall therapies involving *Candida albicans*.

Target	Compound	Methodology	Activity	Ref.
Cell well and hyphal cell wall (Hwp1)	Cinemaldeio encapsulated in lipossome	In vitro: MIC and Biofilm	Cell wall defragmentation and reduction in HWP1 expression, important for adhesion and biofilm formation.	[137]
1,3-β-glucan synthase	Caspofungin-loaded gold nanoparticles	In vitro: MIC	Increased antifungal activity of caspofungin	[139]
1,3-β-glucan synthase	Micafungin-loaded in nanoemulsion	In vitro and in vivo: MIC, Biofilm and *Galleria mellonella* (in vivo assay)	Enhancement of antifungal activity of loaded micafungin and efficient anti-*Candida* albicans activity in *Galleria mellonella* model.	[96]
Cell well	5,11-dimethyl-5*H*-indolo[2,3-*b*]quinoline	In vitro: Biofilms	Increased hexoses; Detrimental effect on the content of pentoses (ribose, xylose and arabinose)	[140]
Chitin and 1,3 β-glucans	*Aucklandia lappa* Decne or *Saussurea lappa*	in vitro: MIC, Sorbitol protection, Spot Assay after Cell Wall Stress on *C. albicans* and calcofluor (for quantification of chitin)	Decrease Chitin and 1,3 β-glucansrease of	[141]
Cell well	LL-37	In vitro: Cell Susceptibility; Protein Secretion and BSA Degradation; Detection of the Redox State of Ero1; Intracellular Reactive Oxygen Species	Cell-wall stress, reactive oxygen species, activation of endoplasmic reticulum-related unfolded protein response signaling, and altered protein secretion.	[142]
Chitin and glucanas	Fleagrass	In vitro	Glucan exposure and chitin increase	[148]
Multi-Target	Methylaervine	In vitro	Cell-wall damage, increased thickness, growth inhibition and filamentation	[149]
1,3-β-glucan synthase	Ibrexafungerp (formerly SCY-078)	In vitro	MIC 0.062 mg/L, MIC range 0.016–0.5 mg/L	[150]
1,3-β-glucan synthase	Ibrexafungerp (formerly SCY-078)	In vitro	MIC ranging from 0.06 to 0.25 µg/mL	[151]
1,3-β-glucan synthase	Ibrexafungerp (formerly SCY-078)	In vitro	MIC from 0.03 mg/L to 0.25 mg/L	
1,3-β-glucan synthase	Enfumafungin MK-3118	Antifungal susceptibility assay with 95 *Candida* strains (20 *C. albicans* and other strains). Determination of IC_50_s wild-type and echinocandin-resistant strains containing fks mutations of *C. albicans* (1 WT and 3 fks mutant strains) and other *Candida* and non-*Candida* strains.	MIC with serum of ≤0.03 µg/mL and MIC without serum of 0.5 µg/mL	[152]
1,3-β-glucan synthase	Rezafungin	In vitro	MIC 0.016 mg/L (0.002–0.125 mg/L)	[153]
1,3-β-glucan synthase	Rezafungin	In vitro	MIC 0.03 to 0.25 in RPMI medium (depend of *Candida*) Activity fungicide	[154]
1,3-β-glucan synthase	Rezafungin	In vitro	MIC_90_ of 0.06 mg/L	[155]
1,3-β-glucan synthase	Rezafungin	Human	Improved quality of life for patients with candidemia.	[156]
1,3-β-glucan synthase	Rezafunin	Human	In the study, all-cause mortality on day 30 was 25.2% and 24.8% for the groups treated with rezafungin and caspofungin, respectively. Mycological eradication after 5 days was higher, at 68.7% and 63.2% for rezafungin and caspofungin, respectively. Overall cure on day 14 was 56.5% and 57.3% for rezafungin and caspofungin, respectively. Finally, safety was 53.3% and 53.7% for rezafungin and caspofungin, respectively.	[158]
glycosylphosphatidylinositol-anchored proteins through inhibition of the fungal enzyme Gwt1	Manogepix/fosmanogepix	In vitro and In vivo	Reduction in fungal load in mice	[159]
14α demethylase	Oteseconazole	Human	Therapeutic cure rate of 66.88% treated with oteseconazole compared to 45.91% with fluconazole. Mycological cure rate of 82.5% with oteseconazole compared to 59.12% treated with fluconazole. Clinical cure rate of 71.25% treated with oteseconazole compared to 55.97% treated with fluconazole.	[161]
Well cells: chitin, β-glucans and structure	Sodium nitroprusside and the alternative oxidase inhibitor salicylhydroxamic acid	In vitro and In vivo	Wall remodeling, increased exposure of chitin and β-glucans and increased immune recognition	[162]
β-d-glucan, chitin and manana	Nicotinamide	In vitro	Exposure to β-glucans, increased chitin and decreased mannans	[164]
Cell well, especially 1,3-β-d-glucan	Cyclohexylidene-4-phenyl-thiazole	In vitro and in vivo	Decreased amount of 1,3-β-d-glucan and inhibition of adhesion.	[165]
recombinant mannosyltransferase 4 (rPmt4p) protein	Anti-rPmt4p monoclonal antibody	In vitro and in vivo	Increased survival of infected mice and increased recruitment of defense cells.	[166]

IC_50_s: half-maximal inhibitory concentration; WT: Wild-type; MIC: Minimum inhibitory concentration.

## Data Availability

All the data is available at work.
